# Effectiveness of synthetic hydroxyapatite versus Persian Gulf coral in an animal model of long bone defect reconstruction

**DOI:** 10.1007/s10195-013-0261-z

**Published:** 2013-08-29

**Authors:** A. Meimandi Parizi, A. Oryan, Z. Shafiei-Sarvestani, A. Bigham-Sadegh

**Affiliations:** 1 Department of Veterinary Surgery and Radiology, School of Veterinary Medicine, Shiraz University, Shiraz, Iran; 2 Department of Veterinary Pathobiology, School of Veterinary Medicine, Shiraz University, Shiraz, Iran; 3 Department of Veterinary Surgery and Radiology, School of Veterinary Medicine, Shahrekord University, Shahrekord, Iran

**Keywords:** Persian Gulf coral, Hydroxyapatite, Radius, Bone healing, Rabbit

## Abstract

**Background:**

There is a continuing search for bone substitutes to avoid or minimize the need for autogenous bone grafts. Hydroxyapatite, a crystalline phase of calcium phosphate found naturally in bone minerals, has shown tremendous promise as a graft material. Coral is an osteoconductive material used as a bone graft extender. This study examined the effect of hydroxyapatite and Persian Gulf coral on osteogenesis in vivo using a rabbit model of bone healing.

**Materials and methods:**

A critical-size defect of 10 mm elongation was created in the radial diaphysis of 36 rabbits and supplied with either hydroxyapatite or coral or left empty (control group). Radiographs of each forelimb were taken postoperatively on day 1 and then at 2, 4, 6, and 8 weeks postinjury to evaluate bone formation, union, and remodeling of the defect. The operated radiuses were removed on the 56th postoperative day and were grossly and histopathologically evaluated. In addition, biomechanical testing was conducted on the operated and normal forelimbs of half of the animals of each group.

**Results:**

In radiological evaluation, bone formation and union were significantly superior in the coral and hydroxyapatite groups in comparison with the control group on the 42nd and 56th day postinjury (*P* < 0.05). There were no statistical differences between groups in remodeling criteria at the 56th day postinjury (*P* > 0.05). In histopathological evaluation, the union scores of the rabbits administered hydroxyapatite or coral were statistically superior to those of the animals of the control group on the 56th day postinjury (*P* < 0.05). In biomechanical evaluation, the control group showed weakness of biomechanical properties in comparison with the coral and hydroxyapatite groups (*P* < 0.05).

**Conclusions:**

According to this study, significant difference was not observed between hydroxyapatite and natural coral and these two materials were significantly better than the control group at 8 weeks postinjury.

## Introduction

There is a continuing search for bone substitutes to avoid or minimize the need for autogenous bone grafts. Autografts are most widely used by surgeons. These grafts contain viable cells such as bone marrow osteoprogenitor cells, collagenous matrix, and noncollagenous extracellular growth and differentiating factors. Consequently, autograft is the preeminent therapy for bone repair, because it is capable of osteogenesis, osteoinduction, and osteoconduction. However, a number of disadvantages such as morbidity at the donor site, the need for general anesthesia or sedation, as well as the occasional need for more than one surgical field have previously been described in application of autografts. In addition, graft survival is unpredictable, its resorption cannot be foretold, and its availability is limited [1, 2]. It is for these reasons that, in recent years, several biocompatible materials have emerged as substitutes for autologous bone. Biocompatible materials can be classified into two major groups: organic and synthetic. Biological biomaterials can be allogeneic or homologous (human cortical bone and demineralized bone matrix or demineralized freeze-dried bone), heterologous, or xenogeneic (organic bovine, porcine, caprine, or coral-derived hydroxyapatite) and replicating (morphogenetic proteins). Among the synthetic biomaterials, application of artificial or synthetic hydroxyapatite, i.e., bioglass and bioceramics, is more common in orthopedic surgery [3].

Recently, bone morphogenetic proteins (BMPs) have been used in clinical trials to enhance bone healing properties [4–6]. It has been stated that BMPs are able to stimulate local undifferentiated mesenchymal cells to transform into osteoblasts (osteoinduction), and lead to early bone formation [7–10]. More study is still necessary to identify which BMPs have greater osteoinductive action and are more efficient in clinical application. Based on recent literature, it seems that bone tissue engineering is the newest option for promoting and accelerating the healing potential of bone defects [11]. In bone tissue engineering, it is possible to combine synthetic scaffolds with biological biomaterials to stimulate cell infiltration and new bone formation, and to enhance the healing process. In this regard, gene therapy (transfer of genes that code growth factors such as BMPs to target cells with the help of a plasmid or viral vector) may provide promising results. However, concern regarding transinfection of the target cell with the gene remains an unresolved issue [12–15].

Stem cells such as adipose-derived stem cells (ASCs) could differentiate into the osteogenic lineage. Furthermore, osteoid matrix formation has been observed when osteoinduced human ASCs were seeded onto hydroxyapatite/tricalcium phosphate scaffolds and implanted subcutaneously in nude mice [16]. Cowan et al. [17] demonstrated that osteoinduced ASCs along with apatite-coated polylactic-coglycolic acid scaffold could repair a critical-sized calvarial defect in a mouse model. Meanwhile, Dudas et al. [18] showed that ASCs in combination with gelatin gel could repair a non-critical-sized defect in a rabbit model with follow-up of 6 weeks. All these results indicate that ASCs could be an alternative cell source for bone engineering [19].

Hydroxyapatite, a crystalline phase of calcium phosphate found naturally in bone minerals, has shown tremendous promise as a graft material. It exhibits initial mechanical rigidity and structure, and demonstrates osteoconductive as well as angiogenic properties in vivo [20]. Additionally, fabricated porous hydroxyapatite scaffolds have been reported to promote strong mechanical interlocking with host bone tissue [20, 21]. Since the extent of bony ingrowth within the scaffold, the functionality of newly regenerative bone tissue, and the development of a vascularized network within the scaffold are dictated by the porous scaffold architecture, extensive studies have been performed to optimize new biomaterials needed for maximal bone tissue integration [22].

Certain coral species form a structure that resembles matrix or bone. Each species builds a structurally and geometrically typical calcium carbonate skeleton. Choice of an appropriate species therefore enables a desired and constant implant structure to be achieved. More than 2,000 coral species have been described from the intertropical area, and, of these, 14 have been studied as possible bone substitutes. The following genera have already been used as bone grafts: *Pocillopora*, *Acropora*, *Montipora*, *Porites*, *Goniopora*, *Fungia*, *Polyphyllia*, *Favites*, *Acanthastrea*, *Lobophyllia*, and *Turbinaria* [23]. The most prominent species were *Porites lutea* and *P. compressa* from the Persian Gulf and Kish Island. The porosity of the skeleton is around 50 %, and the mean size of the pores is 150 μm, with the pores interconnecting with each other [24]. Calcium carbonate (CaCO_3_) resembles hydroxyapatite in many respects. This material is biocompatible and osteoconductive but, like hydroxyapatite, has no osteoinductive properties [25]. The main difference between CaCO_3_ and hydroxyapatite is the resorption rate. Resorption seems to be clinically unimportant with hydroxyapatite, but animal experiments have shown resorption times of only a few weeks when calcium carbonate is used [26]. Therefore, the aim of the present study is to evaluate the effects of Persian Gulf coral and hydroxyapatite on long bone healing processes. The experiment was designed to compare the healing potential of Persian Gulf coral with that of hydroxyapatite, or a defect left empty.

## Materials and methods

### Animals and operative procedures

Thirty-six New Zealand white rabbits (12 months old, mixed sex, weight 2.0 ± 0.5 kg) were kept in separate cages, fed a standard diet, and allowed to move freely during the study. The animals were randomly divided into three equal groups as coral group (*n* = 12), hydroxyapatite group (*n* = 12), and empty group (*n* = 12, control group). All animals were anesthetized by intramuscular administration of 40 mg/kg ketamine hydrochloride and 5 mg/kg xylazine. The right forelimb in all animals was prepared aseptically for operation. A 5-cm skin incision was made craniomedially over the forelimb, and the radius was exposed by dissecting the surrounding muscles. A 10-mm segmental defect was then created in the middle portion of each radius as a critical-size bone defect. The defect of the animals in the coral group was filled with Persian Gulf coral segments. In the hydroxyapatite group, the bone defect was filled with hydroxyapatite segments (OS Satura^®^; Isotis Co., The Netherlands), while the defects of the animals of the control group were left empty. The animals were housed in compliance with our institution’s guiding principles for the care and use of animals. The local Ethics Committee for animal experiments approved the design of the experiment.

## Preparation of coral implants

Coral exoskeleton from *Porites* sp. (Kish Island, Persian Gulf, Iran) was used in the form of cylindrical blocks 2 mm in diameter and 3 mm long. The coral implants were sterilized by autoclaving, which did not affect the composition [27]. The implants were shaped into a cylindrical segmented shape to allow them to fill the created defects.

## Postoperative evaluations

### Radiological evaluation

To evaluate bone formation, union, and remodeling of the defect, radiographs of each forelimb were taken postoperatively on day 1 and then at 2, 4, 6, and 8 weeks postinjury. The results were scored using the modified Lane and Sandhu scoring system [28] (Table 1).

**Table 1:** Modified Lane and Sandhu radiological scoring system

Bone formation	
No evidence of bone formation	0
Bone formation occupying 25 % of the defect	1
Bone formation occupying 50 % of the defect	2
Bone formation occupying 75 % of the defect	3
Bone formation occupying 100 % of the defect	4
Union (proximal and distal evaluated separately)	
No union	0
Possible union	1
Radiographic union	2
Remodeling	
No evidence of remodeling	0
Remodeling of medullary canal	1
Full remodeling of cortex	2
Total points possible per category	
Bone formation	4
Proximal union	2
Distal union	2
Remodeling	2
Maximum score	10

### Gross evaluation

The operated radial bones were removed on the 56th postoperative day; at this time, the operated radius was evaluated for gross signs of healing. Examination and blinded scoring of the specimens included presence of bridging bone indicating complete union (+3 score), presence of cartilage, soft tissue or cracks within the defect indicating possible unstable union (+1 or +2 score), or complete instability at the defect site indicating no union (0 score).

## Histopathological evaluation

Eight weeks after operation, the rabbits were euthanized for histopathological and biomechanical evaluation. The histopathological evaluation was carried out on six rabbits chosen randomly from each group. The right forelimb of each animal was harvested and dissected free of soft tissues. Sagittal sections containing the defect were cut with a slow-speed saw. Each slice was then fixed in 10 % neutral buffered formalin. The formalin-fixed bone samples were decalcified in 15 % buffered formic acid solution and processed for routine histological examination. Two 5-μm-thick sections were cut from the centers of each specimen and stained with hematoxylin and eosin. The sections were blindly evaluated and scored by two pathologists according to the Emery scoring system [29], and based on this scoring system the defects were evaluated as follows: gap empty (score 0), filled with fibrous connective tissue only (score 1), more fibrous tissue than fibrocartilage (score 2), more fibrocartilage than fibrous tissue (score 3), fibrocartilage only (score 4), more fibrocartilage than bone (score 5), more bone than fibrocartilage (score 6), and filled only with bone (score 7).

### Biomechanical evaluation

Biomechanical testing was conducted on the injured and normal contralateral bones of half of the rabbits of each group. The tests were performed using a universal tensile testing machine (Instron, London, UK) [30–32]. The three-point bending test was performed to determine the mechanical properties of the bones. The bones were placed horizontally on two rounded supporting bars located at a separation of 30 mm, and were loaded at the midpoint of the diaphysis by lowering a third bar so that the defect was in the middle and at equal distance from each grip. The bones were loaded at a rate of 10 mm/min until fracture occurred. The behavior of each specimen under loading was characterized by determining the following parameters from the load deformation to destruction curve:

1. Tan *α*: the coefficient of inclination for the linear portion of the load–deformation curve represents the index of stiffness of the material, expressed in N/mm. It is easily calculated by measuring the slope of a line drawn tangent to the curve at any defined point. The slope gives the approximate stiffness of the preparation.
2. Ultimate strength: the highest registered load (N).
3. The specimen’s extension at the ultimate strength region. The term “strain” means the fractional increase in length of the material due to an applied load. It is calculated by dividing the extension by the original length of the specimen. Strain is more useful than extension, because it minimizes the influence of length measurement error and does not depend on the specimen size.
4. Stress: the ultimate strength divided by the cross-sectional area.

The data derived from the load–deformation and stress–strain curves were expressed as mean ± standard error on the mean (SEM) for each group, and the maximum load, stiffness, stress, and strain were measured and recorded.

### Statistical analysis

The radiological, clinical, and histopathological data were compared by Kruskal–Wallis, nonparametric analysis of variance (ANOVA). When *P* values were found to be <0.05, pairwise group comparisons were performed by Mann–Whitney *U* test. The biomechanical data were compared by Student’s *t* test between the treated and normal limb data, and one-way ANOVA test was used for biomechanical analysis between the treated bones of all groups (SPSS version 17 for Windows; SPSS Inc., Chicago, USA).

## Results and discussion

### Radiological findings

**Fig. 1:**
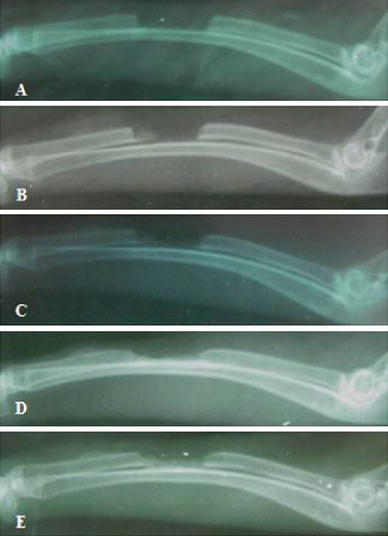
Radiographs of treated forelimb in control group on postoperative day 1 (**a**), 14 (**b**), 28 (**c**), 42 (**d**), and 56 (**e**)

**Fig. 2:**
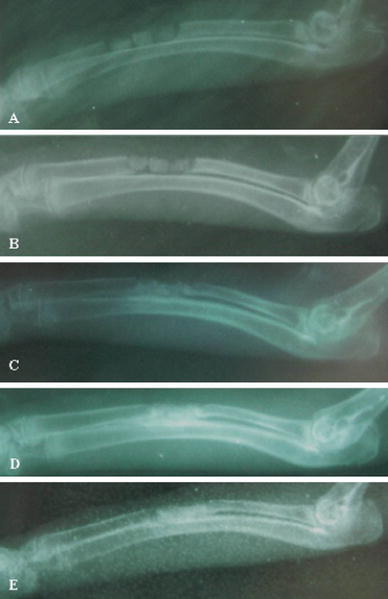
Radiographs of treated forelimb in hydroxyapatite group on postoperative day 1 (**a**), 14 (**b**), 28 (**c**), 42 (**d**), and 56 (**e**)

**Fig. 3:**
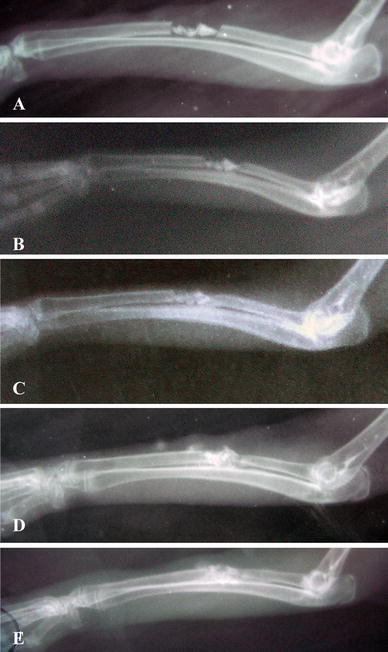
Radiographs of treated forelimb in coral group on postoperative day 1 (**a**), 14 (**b**), 28 (**c**), 42 (**d**), and 56 (**e**)

There was significant difference in bone formation between the defects in the animals of the control group versus those of the coral and hydroxyapatite groups on the 42nd and 56th day postinjury (*P* < 0.05). By day 42 and 56, there was 50–75 % bone formation in the defects of the animals of the coral group, 75–100 % bone formation in the animals of the hydroxyapatite group, and 25–50 % bone formation in those of the control group (Table 2; Figs. 1, 2, 3).

**Table 2:** Radiographical findings for bone formation at various postoperative intervals

Postoperative days	Median (min–max)			*P* ^a^
	Control (*n* = 12)	Coral (*n* = 12)	Hydroxyapatite (*n* = 12)	
14	0 (0–1)	0 (0–0)	0 (0–0)	0.1
28	1 (0–1)	1 (1–2)	1 (0–2)	0.06
42	1 (0–3)	2 (1–3)^b^	2 (1–3)^c^	**0.04**
56	2 (1–3)	3 (2–3)^d^	3 (2–4)^e^	**0.05**

Significant *P* values are presented in bold

^a^Kruskal–Wallis nonparametric ANOVA

^b^*P* = 0.03 (compared with control by Mann–Whitney *U* test)

^c^*P* = 0.04 (compared with control by Mann–Whitney *U* test)

^d^*P* = 0.02 (compared with control by Mann–Whitney *U* test)

^e^*P* = 0.01 (compared with control by Mann–Whitney *U* test)

**Table 3:** Radiographical findings for proximal union at various postoperative intervals

Postoperative days	Median (min–max)			*P* ^a^
	Control (*n* = 12)	Coral (*n* = 12)	Hydroxyapatite (*n* = 12)	
14	0 (0–0)	1 (0–0)	0 (0–0)	1.0
28	1 (0–1)	1 (0–1)	1 (0–1)	0.5
42	1 (0–1)	1 (1–2)^b^	1 (1–2)^c^	**0.05**
56	1 (0–2)	2 (1–2)^d^	2 (1–2)^e^	**0.01**

Significant *P* values are presented in bold

^a^Kruskal–Wallis nonparametric ANOVA

^b^*P* = 0.02 (compared with control by Mann–Whitney *U* test)

^d^*P* = 0.05 (compared with control by Mann–Whitney *U* test)

^c^*P* = 0.02 (compared with control by Mann–Whitney *U* test)

^e^*P* = 0.01 (compared with control by Mann–Whitney *U* test)

Bone union had occurred in the rabbits of the hydroxyapatite and coral groups by day 42 and 56 postsurgery, but not in the animals of the control group. In addition, bone union in the animals of the hydroxyapatite and coral groups by day 42 and 56 postsurgery was more prominent than in the control group. This trend continued, with less union occurring in the animals of the control group (Tables 3, 4; Figs. 1, 2, 3).

**Table 4:** Radiographical findings for distal union at various postoperative intervals

Postoperative days	Median (min–max)			*P* ^a^
	Control (*n* = 12)	Coral (*n* = 12)	Hydroxyapatite (*n* = 12)	
14	0 (0–1)	0 (0–0)	0 (0–0)	0.1
28	1 (0–1)	1 (0–1)	1 (0–1)	0.5
42	2 (0–2)	1 (0–2)	1 (0–2)	0.5
56	2 (0–2)	2 (1–2)	1 (1–2)	0.1

Significant *P* values are presented in bold

^a^Kruskal–Wallis nonparametric ANOVA

There were no statistical differences between groups. The animals of the hydroxyapatite group showed better remodeling criteria on day 56 than those of the control group, although statistical analysis did not show any significant differences (Table 5; Figs. 1, 2, 3).

**Table 5:** Radiographical findings for remodeling at various postoperative intervals

Postoperative days	Median (min–max)			*P* ^a^
	Control (*n* = 12)	Coral (*n* = 12)	Hydroxyapatite (*n* = 12)	
14	0 (0–0)	0 (0–0)	0 (0–0)	1.0
28	0 (0–0)	0 (0–0)	0 (0–0)	0.4
42	0 (0–0)	0 (0–0)	0 (0–0)	1.0
56	0 (0–1)	1 (0–1)	1 (0–2)	0.1

Significant *P* values are presented in bold

^a^Kruskal–Wallis nonparametric ANOVA

### Gross and histopathological findings

The defect areas of the rabbits of both treated groups showed various amounts of new bone formation; however, the bone defects of the control group left blank or generally contained the least amounts of new bone and were often filled with a mixture of fibrous connective tissue and cartilage. The union scores of the rabbits administered hydroxyapatite or coral were statistically superior to those of the animals of the control group (Table 6). The union scores at macroscopic level correlated closely with the radiographic union scores on day 56 postinjury.

**Table 6:** Bone measurements at macroscopic and microscopic level

Bone evaluation type	Median (min–max)			*P* ^a^
	Control (*n* = 6)	Coral (*n* = 6)	Hydroxyapatite (*n* = 6)	
Macroscopic union^*^	1 (1–2)	2 (1–3)^b^	2 (2–3)^c^	**0.00**
Microscopic evaluation^†^	2 (1–5)	6 (5–7)^d^	6 (5–7)^e^	**0.003**

Significant *P* values are presented in bold

^*^Complete union (+3 score), presence of cartilage, soft tissue or cracks within the defect indicating possible unstable union (+1 or +2 score), complete instability at the defect site indicating nonunion (0 score)

^†^Empty (0 score), fibrous tissue only (1 score), more fibrous tissue than fibrocartilage (2 score), more fibrocartilage than fibrous tissue (3 score), fibrocartilage only (4 score), more fibrocartilage than bone (5 score), more bone than fibrocartilage (6 score), bone only (7 score)

^a^Kruskal–Wallis nonparametric ANOVA

^b^*P* = 0.005 (compared with control by Mann–Whitney *U* test)

^c^*P* = 0.005 (compared with control by Mann–Whitney *U* test)

^d^*P* = 0.001 (compared with control by Mann–Whitney *U* test)

^e^*P* = 0.0 (compared with control by Mann–Whitney *U* test)

**Fig. 4:**
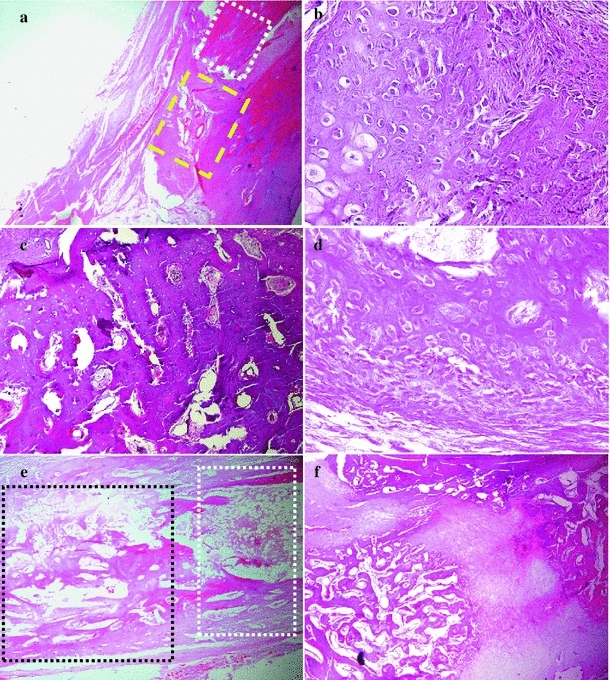
Photomicrographs from the control group showing fibrous connective tissue in the defect area without bone marrow formation (*yellow rectangle*), old bone region (*white rectangle*) (**a**, H & E stain 4×), and extensive fibrocartilage (**b**, H & E stain 40×). Photomicrographs from the hydroxyapatite group, showing trabecular bone formation (**c**, H & E stain 10×) and woven bone (**d**, H & E stain 40×). Photomicrographs from the coral group showing trabecular-pattern bone formation in grafted area (*black rectangle*) and grafted area with old bone and marrow (*white rectangle*) (**e**, H & E stain 4×). Note the trabecular bone and chondroplasia zone in the coral group (**f**, H & E stain 4×)

At the histopathologic level, the defects of the animals of the hydroxyapatite and coral groups showed more advanced healing criteria than those of the control group (Table 6). Fibrous nonunion or fibrocartilage in the defects of the animals of the control group was dominant, and the lesions of these animals showed poor revascularization. Bridging callus or histological union did not develop in any of these defects. These criteria led to a very slow healing process in the animals of the control group (Fig. 4).

The defects of two rabbits of the coral group were filled with mature cortical bone, and the lesions in the remaining four rabbits were substituted by fibrocartilage tissues. Although the defects of the animals in the coral group showed some angiogenic activity, the neovascularization was not as good as in the hydroxyapatite group (Fig. 4).

Normal trabecular and woven bone were uniformly formed within the defects of the animals treated with the hydroxyapatite regimen, and the lesions of this group were filled with woven bone and showed proper maturation; however, the defect of one rabbit contained more fibrocartilage than bone. The regenerated bone completely spanned the defect and most histologic union was occurred. Active endochondral ossification and secondary fracture repair took place in the middle of the defects of the animals of the hydroxyapatite and coral groups (Fig. 4). No significant inflammatory response was evident in the lesions of the animals of the different groups at 8 weeks postinjury, although it may have been present earlier.

### Biomechanical findings

There was significant difference between the injured versus normal bone of the control group in terms of ultimate strength (*P* = 0.01) and stiffness (*P* = 0.04), and the normal bones had superior ultimate strength and stiffness compared with their normal contralaterals. However, the ultimate strength in the treated animals of the hydroxyapatite group showed more advanced values that were not statistically significantly different from those of their normal contralaterals (Table 7).

**Table 7:** Biomechanical findings at 56th postoperative day

Three-point bending test criteria	Mean ± SEM					
	Control (*n* = 6)		Coral (*n* = 6)		Hydroxyapatite (*n* = 6)	
	Normal limb	Treated limb	Normal limb	Treated limb	Normal limb	Treated limb
Ultimate strength (N)	66.8 ± 10.5^a^	38.6 ± 7.5	63.6 ± 14.5	53.16 ± 9.5	60.6 ± 10.5	70.8 ± 8.4^b^
Stress (N/mm^2^)	3.64 ± 0.7	2.18 ± 0.3	3.49 ± 1.1	2.43 ± 0.43	4.1 ± 0.83	3.75 ± 0.71
Stiffness (N/mm)	128.3 ± 7.4^c^	91.6 ± 14.9	133.3 ± 13.5	88.0 ± 14.9	96.0 ± 11.6	75.0 ± 5.6
Strain (%)	7.9 ± 0.5	8.4 ± 0.6	8.35 ± 0.7	9.3 ± 0.84	7.1 ± 1.1	6.7 ± 0.80

^a^*P* = 0.01 (normal limb compared with treated limb in control group by Student *t* test)

^b^*P* = 0.05 (treated limb compared with treated limb in control group by one-way ANOVA test)

^c^*P* = 0.04 (normal limb compared with treated limb in control group by Student *t* test)

The objective of this study was to evaluate healing of critical-size radial bone defects treated with hydroxyapatite or natural coral in comparison with a control (empty) group. The radial bone defect of rabbits is a convenient model for study of bone-regenerative materials because of its lack of fixation requirements [33]. Segmental defects as long as 10 mm were created in the middle portion of the radius to induce nonunion defect and prevent spontaneous and rapid healing [34].

Autogenous bone still remains the “gold standard” of bone graft material in all facets of orthopedic surgery and is commonly used as a standard against which allografts and graft substitutes are compared [35–40]. They may provide a source of osteoprogenitor cells (osteogenesis), induce formation of osteoprogenitor cells from surrounding tissues (osteoinduction), and provide mechanical support for vascular and bone ingrowth (osteoconduction) [41]. In our study we used three groups for comparison, but it seems that we should have included another group with autogenous bone grafting as a positive control group. However, hydroxyapatite and coral materials act solely as osteoconductive materials and have no osteoinductive properties [20, 25]. These different properties led to the three-group comparison design of our study, and we did not include autograft as an additional group. There are a wide range of biomaterials that could be used as bone substitutes, depending on their bioactivity. Use of calcium phosphate ceramics as implant materials is common, and previous studies [42, 43] indicated that hydroxyapatite (HA) implanted into osseous surgical defects at various sites does not elicit an inflammatory response and is essentially nonresorbable. It has also been shown that HA allows physiologic contouring of a treated site, while it may or may not allow incorporation of bony ingrowth [42, 44, 45]. Clinically, coral has been successfully used in spinal fusion [46, 47], cranial surgery [48], and dentistry [49]. It is osteoconductive but not osteogenic.

Based on the four outcome measures described in this study, it was observed that defects grafted with hydroxyapatite or natural coral showed significantly more bone formation than the negative control (empty defect) at 8 weeks.

Hydroxyapatite, a crystalline phase of calcium phosphate found naturally in bone minerals, has shown tremendous promise as a graft material. It exhibits initial mechanical rigidity and structure, and demonstrates osteoconductive as well as angiogenic properties in vivo [20, 50, 51]. In osteoperiosteal gaps bridged with hydroxyapatite only, the porosities were invaded with fibrous tissue or fibrocartilage tissues and the defects were not filled with bone tissue. Occasionally, bone formation was observed in direct contact with hydroxyapatite, confirming its osteoconductive ability, albeit insufficient to enable union. These findings are similar to those reported using hydroxyapatite. When the gap reaches a critical size, the osteoconductive properties of the material are insufficient to fill the gap with formation of new bone [52].

More unexpected was the formation of cortex and medullary canal together with mature lamellar bone observed in most of the cases. Previous in vitro studies showed that artificial bone graft materials support attachment, growth, and differentiation of bone marrow stromal cells [53]. The findings of the present study suggest that hydroxyapatite is a suitable material in vivo. It serves as a template to guide bone morphogenesis in a clinically relevant volume.

According to this study, significant difference was not observed between hydroxyapatite and natural coral, and these two materials led to bone formation in a similar way. It has been shown previously that natural coral (CaCO_3_) resembles hydroxyapatite in many aspects. The material is biocompatible and osteoconductive but, similar to hydroxyapatite, has no osteoinductive properties [12]. The main difference between coral and hydroxyapatite is its chemical structure, as hydroxyapatite is calcium phosphate whereas coral is calcium carbonate [25, 26, 54]. In addition, a study by Mora et al. [55] that compared natural coral skeleton versus porous hydroxyapatite for treating periodontal bone defects in human subjects found no significant difference between the use of coral skeleton and porous hydroxyapatite for bony defect filling, and statistical analysis revealed the beneficial effects of using each biomaterial.

The biomechanical evaluation performed in this study indicated initial failure at the interosseous membrane, suggesting a strong load-sharing mechanism through this syndesmosis between the radius and ulna. The syndesmosis was shown to have extensive calcification, accounting for a large fraction of the bone volume in the defect and possibly contributing to the bone ingrowth into the scaffold. This was supported by both histopathologic and radiographic evidence showing new bone growth in a cone-like fashion and from the direction of the interosseous membrane in defects implanted with scaffolds as well as in defects with no treatment. Thus, separating the radius from the ulna for biomechanical testing may damage this tissue. It is also important to consider that the radius and ulna act as a unit in the physiological setting and that it may be more biologically relevant to evaluate them together [56].

Based on the radiological, histopathological, and biomechanical findings of the present study, healing of defects in animals of the control group was not very efficient and the defect area was filled with fibrous tissues and rarely with cartilage instead of osseous tissue. Barnes et al. [57] indicated that chondrocytes derived from mesenchymal progenitors proliferate and synthesize cartilaginous matrix until all fibrinous/granulation tissue is replaced by cartilage. Where cartilage production is deficient, fibroblasts replace the region with generalized fibrous tissue. Discrete cartilaginous regions progressively grow and merge to produce a central fibrocartilaginous plug between the fractured fragments that splints the fracture. Overall, this study demonstrates that both hydroxyapatite and Persian Gulf coral showed significantly more bone formation than the negative control (empty defect) at 8 weeks after surgical operation.
